# The Use of MALDI-TOF MS as a Diagnostic Tool for Adult *Trichuris* Species

**DOI:** 10.3389/fvets.2022.867919

**Published:** 2022-05-11

**Authors:** Julia Rivero, Antonio Zurita, Cristina Cutillas, Rocío Callejón

**Affiliations:** Department of Microbiology and Parasitology, Faculty of Pharmacy, University of Seville, Seville, Spain

**Keywords:** *Trichuris*, MALDI-TOF MS, diagnosis, nematode, internal database

## Abstract

Trichuriasis is considered a neglected tropical disease, being the second most common helminthiasis in humans. Detection of *Trichuris* in routine diagnosis is usually done by microscopic detection of eggs in fecal samples. Other molecular analyses are more reliable and could be used, but these analyses are not routinely available in clinical microbiology laboratories. The use of matrix-assisted laser desorption/ionization-time of flight (MALDI-TOF) mass spectrometry (MS) is increasing since the last decades due to its recent evidence as a potential role for reliable identification of microorganisms and a few nematodes. But, for parasites detection, normalized protocols and the acquisition and introduction of new species to the database are required. We carried out a preliminary study confirming the usefulness of MALDI-TOF MS for the rapid and reliable identification of *Trichuris suis* used as control and the creation of an internal database. To create main spectra profiles (MSPs), the different parts of five whipworms (esophagus and intestine) were used, developing different tests to verify the repeatability and reproducibility of the spectra. Thus, to validate the new internal database, 20 whipworms, separating the esophagus and intestine, were used, of which 100% were accurately identified as *T. suis*, but could not distinguish between both parts of the worm. Log score values ranged between 1.84 and 2.36, meaning a high-quality identification. The results confirmed that MALDI-TOF MS was able to identify *Trichuris* species. Additionally, a MALDI-TOF MS profile of *T. suis* proteome was carried out to develop the first internal database of spectra for the diagnosis of trichuriasis and other *Trichuris* spp.

## Introduction

Helminths constitute one of the most common, ecologically diverse, and speciose animal groups in the world. Several species of nematodes are of huge economic importance or medical interest. The most important species of soil-transmitted helminth (STH) infections, infecting humans are *Ascaris lumbricoides* (roundworm), *Trichuris trichiura* (whipworm) and *Necator americanus* and *Ancylostoma duodenale* (hookworms). Nearly 24% of the world's population is infected with soil-borne helminths (1). The prevalence is higher in marginalized populations in the tropics and subtropics, where there is a lack of basic sanitation services (2).

*T. trichiura* is a nematode, which is the etiological agent of the parasitic disease known as “trichuriasis.” Trichuriasis is considered as a neglected tropical disease and has a worldwide geographical distribution. *T. trichiura* is the second most common helminth in humans. Moreover, whipworms are among the most common intestinal parasites of humans and animals, causing significant diseases and economic losses globally (3, 4). Whipworms can be found in a large range of hosts, in addition to humans (*T. trichiura*), in suids (*T. suis*), sheep, goats, and bovines (*T. ovis* and *Trichuris discolor*), dogs (*Trichuris vulpis*), non-human primates (NHP) (*Trichuris* spp.) and several putative new *Trichuris* species (5–10).

Traditionally, the identification of *Trichuris* species was carried out according to the host where the whipworm was found, and later, by studies based on morphological and biometrical features of adults. But due to the phenotypic plasticity of these parasites (host-induced variation, lack of morphological characteristics, and overlap of morphological characteristics and biometrical data between species), it is highly difficult to distinguish between closely related *Trichuris* species (11–14). Hence, molecular studies, such as polymerase chain reaction (PCR) and sequencing, are used as a tool to differentiate species (5–7, 9, 10, 15–20). While the morphological identification is a rapid and less costly procedure, the related *Trichuris* species are hardly morphologically distinguishable. Moreover, the diagnosis of *Trichuris* is made by observing eggs in fecal samples and requires qualified personnel. Thus, the improvement of an accurate, fast, less expensive, and more accessible diagnostic technique for the identification of parasites would be desirable.

The matrix-assisted laser desorption/ionization time-of-flight mass spectrometry (MALDI-TOF MS) has been commonly introduced as a diagnostic method in laboratories, that analyzed complex molecules, such as proteins, by producing protein fingerprint signatures (spectra) from proteins extracts of organisms (21). The creation of reference spectra database by the acquisition of spectra has been used to identify species of parasites (22–25). MALDI-TOF MS has been suggested as a rapid and reliable identification technique of bacteria (26, 27). Lately, authors have demonstrated that MALDI Biotyper software can be used, in addition to bacteria (28–31), mycobacteria (28, 32), fungi (31), and most recently, in viruses (33), protozoans, arthropods (22, 34, 35), and a few nematodes (24, 36–40).

MALDI-TOF MS has revealed many advantages compared with other diagnostic tools (such as PCR assays). When the mass spectrometer and the corresponding databases are available in a laboratory, the identification is inexpensive, and the sample preparation procedure does neither require highly skilled technicians or complex additional laboratory infrastructure (41–43). Other advantages are that MALDI-TOF MS is significantly less susceptible to contamination, since the samples do not require special collected and preserved conditions, and the results are available within a few minutes. Nevertheless, the constant power supply is a limitation for the suitability of the technique in resource-limited environments. However, MALDI-TOF MS is more and more available in reference laboratories around the world including in developing countries (41–45). Furthermore, recent studies suggested that MALDI-TOF MS technique is of great importance due to its applicability in the discovery of antibiotic resistance in microorganisms, disinfectants, and the production of toxins from pathogens (29, 46–48).

Recently, a review about the use of MALDI-TOF in human and veterinary helminthology confirms that this technique is reliable and reproducible for nematode parasites. They suggested that it is necessary for many studies for more different species of nematodes, especially in common nematode parasites in the world, and to create a single database with all nematode species. And also, add the necessary spectra to the internal o commercial database to be able to identify larvae and eggs to open the possibility to analyze fecal samples by MALDI-TOF MS and obtain a fast and reliable identification and diagnosis (49).

Based on this background, the aim of this study was to evaluate the usefulness of MALDI-TOF MS as an effective diagnostic tool for the specific identification of *Trichuris* species. For this purpose (i) a standard protocol for the extraction of proteins was developed using *Trichuris suis* as control, (ii) a preliminary specific reference spectra database was created characterizing this species, *T. suis*, (iii) the standardized protocol of *T. suis* was validated for more species of *Trichuris* using MALDI-TOF MS analysis: *Trichuris* sp. from *Hystrix cristata, T. trichiura* from *Macaca sylvanus, T. vulpis* from *Canis lupus familiaris* and *T. ovis* from *Capra hircus* collected on Spain, (iv) an MS reference spectra in-house database for *Trichuris* species-specific identification was created based on molecular *Trichuris* identification comparatively with the new species of *Trichuris* analyzed, and (v) an analysis of the results obtained by MALDI-TOF MS was performed to verify the usefulness of MALDI-TOF MS in the phylogenetic and taxonomic study of *Trichuris* species.

## Materials and Methods

### Ethics Statement

This study does not require approval by the ethics committee. Adult *Trichuris* samples were obtained from their caecum postmortem. The specimens of *T. suis* and *T. ovis* were obtained through a slaughterhouse; in a zoo, for adults of *T. trichiura* from *M. sylvanus* and *Trichuris* sp. from *H. cristata*, and in groups of dogs, for adults of *T. vulpis*, in strict accordance with good animal practices.

### Sample Collection

For this study, whipworms belonging to the species *T. suis* from swine (*Sus scrofa domestica*) and *T. ovis* from goats (*Capra hircus*) were collected in slaughterhouses in the provinces of Seville and Huelva, and Seville (Spain), respectively. Specimens identified as *Trichuris* sp. from *H. cristata* and *T. trichiura* from *M. sylvanus* were collected from Bioparc Fuengirola (Spain), and Zoo Castellar (Spain), respectively. Finally, *T. vulpis* adults were collected from dogs (*Canis lupus familiaris*) in the provinces of Seville and Huelva. Separately, whipworm samples were washed several times in saline solution (0.9% w/v), and then frozen at −20°C until posterior analysis.

### Sample Preparation and Morphological Identification

Adult worms were defrosted and dried at room temperature. The morphological identification of different *Trichuris* species was carried out according to previous studies (15, 18, 20, 50–52). Nonetheless, morphologically the different species of *Trichuris* are difficult to differentiate. Thus, a molecular study was carried out.

Then, two different samples of the *T. suis* adults (esophagus and intestine) were used separately for MALDI-TOF MS analysis to check which part of the worm gave greater reliability and easier reproducibility.

For this reason, the preliminary study with only adults of *T. suis* was performed using 31 *T. suis* worms, using the esophagus and the intestine of adults as samples. Further, these two samples were assayed in the rest of the species of *Trichuris* for proteomic and molecular analysis, respectively.

### Molecular Analysis

#### DNA Extraction, PCR, and Sequencing

To verify the accuracy of the species identification of the samples collected for MALDI-TOF MS, the morphological studies were corroborated with molecular analysis. The total genomic DNA from intestine worms was extracted using DNeasy Blood and Tissue Kit (Qiagen; Hilden, Germany) according to the manufacturer's instructions. The quality of extractions was assessed using 0.8% agarose gel electrophoresis infused with SYBR® Safe DNA gel stain (Thermo Fisher Scientific, MA, USA).

The *cytochrome* b (*cyt*b) mitochondrial DNA (mtDNA) gene of two to five intestines DNA samples of each *Trichuris* species (*T. suis, Trichuris* sp. from *H. cristata, T. trichiura, T. vulpis*, and *T. ovis*), were amplified by standard PCR by a thermal cycler (Eppendorf AG; Hamburg, Germany), while the esophagus was reserved for MALDI-TOF MS. The forward primer utilized was D769 (5′-GAGTAATTTTTATAATRCGRGAAGT-3′) (53) and the reverse primer utilized was D770 (5′- AATTTTCAGGRTCTCTRCTTCAATA-3′) (53), and the following PCR mix: 5 μl each primer (10 μM), 25 μl GoTaq G2 Green Master Mix, 5 μl template DNA, and nuclease-free water to 50 μl. The following conditions were applied: 94°C at 5 min (denaturing); 36 cycles at 94°C at 30 s (denaturing), 50°C at 30 s (annealing), and 72°C at 30 s (primer extension); followed by 7 min at 72°C (final extension). The PCR products were visualized on SYBR Safe stained with 2% w/v Tris–Borate–EDTA (TBE) agarose gels. Then, bands were eluted and purified using the Wizard SV Gel and PCR Clean-Up System Kit (Promega, WI, USA). Once purified and concentrated, the PCR products were sequenced in both directions by Stab Vida (Lisbon, Portugal).

#### Sequence Analysis Species Identification

The forward and reverse sequences obtained were analyzed using Multiple Sequence Alignment by CLUSTALW, to generate a consensus sequence for each specimen. The sequences were compared with sequences available in the National Center for Biotechnology Information (NCBI) GenBank database using the Basic Local Alignment Search Tool (BLASTn) algorithm for identification (https://blast.ncbi.nlm.nih.gov/Blast.cgi). Sequences obtained were submitted to the NCBI GenBank database.

### MALDI-TOF Analysis

#### Preliminary MALDI-TOF Analysis

##### Protein Extraction

For the preliminary study with *T. suis* worms, each worm sample, esophagus, and intestine, separately was placed in a 1.5 ml sterile Eppendorf. Afterward, each sample was pooled with 10 mg zirconia/silica beads (0.5 mm) along with 20–30 μl of a mix of 70% (v/v) formic acid and 50% (v/v) acetonitrile. Then, the samples were homogenized using the TissueLyser II system (Qiagen GmbH) in three cycles of 30 s at a frequency of 30 Hz. The homogenized samples were centrifuged at 10,000 g for 30 s.

##### Target Plate Preparation and Measurements

From each sample, 1 μl of the supernatant was carefully dropped on to the MALDI-TOF target in eight different spots for the creation of the main spectrum profiles (MSPs) (25), and four times for the validation test (54). Air dried and each spot was then recovered with 1 μl of CHCA matrix solution composed of saturated α-cyano-4-hydroxycinnamic acid (Sigma–Aldrich, Co., MO, USA), 50% acetonitrile (v/v), 2.5% trifluoroacetic acid (v/v) (Thermo Scientific, Rockford, IL, USA), and 47.5% (v/v) high-performance liquid chromatography (HPLC) grade water (34). Bacterial test standard (BTS) (Bruker Daltonics, Bremen, Germany) was used to calibrate the machine (an *Escherichia coli* extract), which is spiked with two high molecular weight proteins. The matrix solution was loaded in quadruplicate to control the matrix quality (54). Then, at room temperature, having dried for several minutes, the plate was placed into the Microflex LT Mass Spectrometer (Bruker Daltonics) for MALDI-TOF MS.

##### MALDI-TOF Parameters

The MALDI-TOF MS measurements were carried out on a range of 2,000–20,000 Da, *m*/*z* (mass to charge) and with detection in the linear positive in mode with a laser frequency of 50 Hz, following the calibration with BTS.

For each spot, 240 laser shots were performed in four regions, and the measurements were automatically acquired using the AutoXecute method of the flexControl v3.4 software (Bruker Daltonics; Bremen, Germany). The spectrum profiles (protein mass profiles) were generated and visualized by Flex Analysis v3.3 software and were exported to ClinProTools v2.2 and MALDI-Biotyper v3.1.66 (Bruker Daltonics; Bremen, Germany) for data processing (smoothing, baseline subtraction, and peak picking). The acceleration voltage was 20 kV, and the extraction delay time was 200 ns (54). Concisely, the maximum mass error of each individual spectrum was 2,000 Da, the desired peak frequency minimum was 25% and the desired mass error for the MSP was 200 Da.

##### Spectral Analysis and Preliminary Database Creation

For the creation of species-specific MSPs, according to Diarra et al. (55), 2–6 specimens of each species is enough. Hence, protein extracts of five esophagus from *T. suis* worms and three intestines from *T. suis* worms were spotted on the MALDI-TOF target plate eight times per sample. Then, each spot was measured four times. For each sample worm, this procedure was carried out on two replicates on different days to demonstrate repeatability and the reproducibility analysis. The combination of the results of the spectra from each specimen was used to create MSP by the automated function of the MALDI-Biotyper using the default parameter set of the “Bio Typer MSP Creation Standard Method” (54). The quality of each raw spectra was assessed with Flex Analysis software version 3.4. (Bruker Daltonics; Bremen, Germany). This program was also used to remove all flatlines and outlier peaks and smooth intensities and edit peak changes within spectra whenever they exceed 500 ppm.

##### Validation Test

The recently developed internal database underwent two different validation procedures. Starting with an internal validation, where all the spectra of each group of samples obtained through the MSP creation process were analyzed. And finally, a blind test analysis, in which the samples were measured by MALDI-TOF to evaluate the ability of the database to reliably identify these samples. For the blind test analysis, 20 specimens were analyzed. Protein extract from each sample was spotted on the MALDI-TOF target in quadruplicate. Hence, each sample was associated with four spectra. A total of 72 high-quality spectra from *T. suis*'s esophagus (for two samples the protein extract process failed) and 80 from *T. suis*'s intestine, were selected to query the database. The results obtained against the reference library were shown as log score values (LSVs) for each spectrum. LSV range from 0 to 3 reflects the results of a comprehensive comparison of peak position and intensity between two spectra (LSV from 0 to 1.699: no reliable identification; 1.7 to 1.999: probable genus identification; 2.0 to 2.299: secure genus identification and probable species identification, and 2.300 to 3.000: highly probable species identification) (25, 40).

#### Validation of MALDI-TOF Analysis

The analysis was carried out according to the preliminary study to validate and verify the procedures and the parameters used.

##### Spectral Analysis and Database Creation

For each *Trichuris* species (*Trichuris* sp. from *H. cristata, T. trichiura, T. vulpis*, and *T. ovis*), MSPs were created as previously described by the preliminary protocol. To actualize the internal database, five specimens for *Trichuris* sp. from *H. cristata* and for *T. trichiura* from *M. sylvanus*, two specimens for *T. vulpis*, and four specimens for *T. ovis* were used by running the MALDI Biotyper software automatically.

##### Validation Test

To assess the ability of the database to reliably identify these samples, a blind test was performed. A total of 63 good quality spectra from the four different *Trichuris* species analyzed were selected to examine the updated database. For each specimen, the protein extracted was spotted on the MALDI-TOF target four times, generating four spectra associated with the same sample. The results obtained for the query of the internal database are shown as log score values (LSVs) for each spectrum.

To determine the distances and similarity among MSPs, a hierarchical clustering of the mass spectra was performed, using the spectra utilized for the MSP creation, and the dendrogram function within MALDI Biotyper software.

### Phylogenetic Analysis

To evaluate the similarity among all *cyt*b *Trichuris* sequences, the number of nucleotide and amino acids differences per sequence was calculated using Compute Pairwise Distances based on the number of differences method of MEGA X v10.1.8 (56).

For phylogenetic analysis, two methods—maximum likelihood (ML) and Bayesian inferences (BI)—were used. PhyML 3.0 was used to generate the ML tree (57), and for BI was used MrBayes v.3.2.6 (58). jModelTest to resolve the best-fit substitution model for the nucleotide data set was employed (59). According to Akaike Information Criterion, models of evolution were determined (57, 60). GTR + I + G model, with rate variation along the length of the alignment (+ G) and allowing for a proportion of invariant sites (+ I), was selected for the nucleotide data set. Topology support was examined using bootstrapping (heuristic option) (61) over 1,000 replications to assess the relative reliability of clades. The Bayesian posterior probabilities (BPPs) comprise the percentage converted. To determine if the number of generations completed was sufficient, was used the standard deviation of split frequencies; every 500 generations the chain was sampled, and each dataset was run for 10 million generations. Based on an assessment of convergence, trees from the first million generations were discarded. Examination of the log-likelihood values of the chains is determined empirically during the burn.

## Results

### Preliminary MALDI-TOF Analysis

#### Comparative Analysis of *T. suis* Samples Used for MSP Database Creation (MALDI-TOF Analysis)

As mentioned before, for the creation of different reference spectra, firstly, two distinct body parts (esophagus and intestine) of *T. suis* worms were used. We included five specimens of which five esophagus and three intestines were used. The *cox*1 mtDNA partial sequence valid the specific analysis in the reference samples against previously deposited sequences by BLASTn, revealing approximately a 100% identity between *T. suis* (Supplementary Table S1) and the *T. suis* sequences from this study (reference accession number: OU756954). From the three *T. suis* worm sequences obtained, only 1 haplotype was generated.

**Figure 1:**
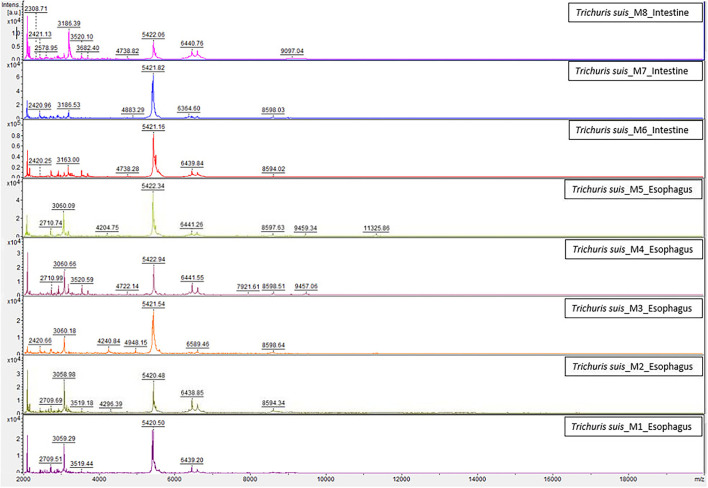
Comparison of the spectra created for the internal database between esophagus and intestine samples of *T. suis* worms by MALDI-TOF MS. a.u., arbitrary units; *m*/*z*, mass-to-charge ratio (Da).

**Figure 2:**
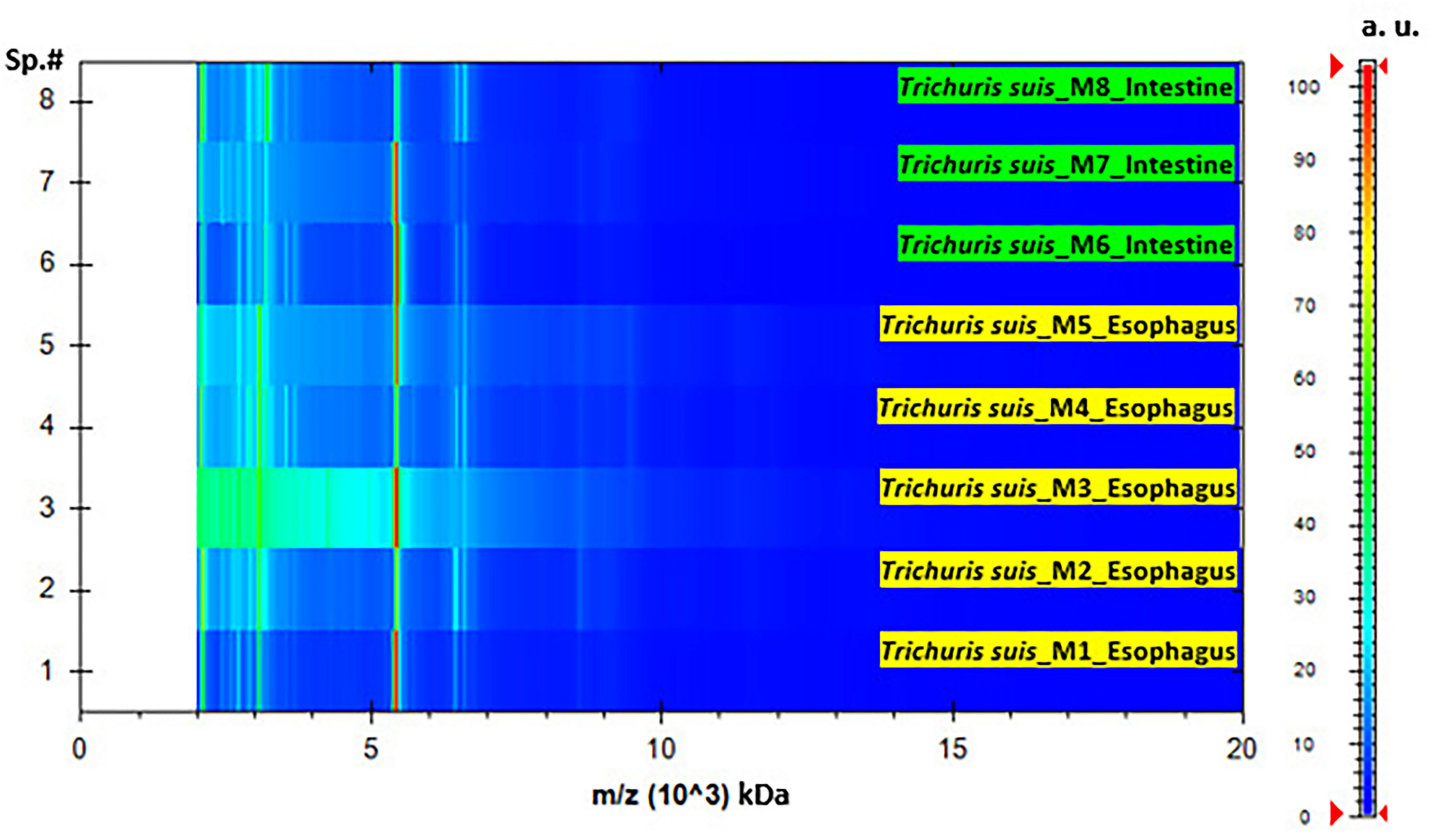
Pseudo-gel representing the protein profile obtained after MALDI-TOF MS analysis of *T. suis* specimens' representative of esophagus and intestine samples. On the *x*-axis, the mass-to-charge values (*m*/*z*, kDa) are reported, at the same time, on the right *y*-axis, the scale bar reveal the relationship between the color intensity and the peak intensity, expressed by arbitrary units (a.u.). The spectra samples are represented on the left *y*-axis (Sp.#).

The homogenization and sample preparation protocol described above provided high-quality spectra (Figure 1). Both, results based on esophagus and intestines samples showed similar spectra, with profiles of high intensity and strongest peaks placed in the same range, however, peaks intensity in the esophagus samples was slightly higher. The reference MSPs obtained for each type of sample by MALDI-TOF with high-intensity peaks in the 2–20 kDa range are presented in Figure 1. The highest density peaks were in the region comprised between 2 and 9.5 kDa, with clusters of signals in ranges corresponding to 2.1–2.3, 3–3.3, 5.2–5.6, and 6.2–6.6 kDa (Figure 2). There were no significant differences between esophagus and intestine samples of *T. suis*.

#### Internal Database Creation

All MSPs obtained were analyzed with the commercially common database using FlexAnalysis software for bacteria and fungi identification and reliable identification was not achieved with all LSVs <1.7. The posterior analysis of the raw spectra obtained during MSP creation, which used a combination of the commercial and internal database, revealed the identification of *T. suis* in all the samples, but it was not possible to differentiate between esophagus and intestines of the same *T. suis* species. LSVs of *T. suis* esophagus sample ranging from 2.43 to 2.82, identified as *T. suis* esophagus, and from 2.10 to 2.27 identified as *T. suis* intestine. LSVs of *T. suis* intestine samples ranged from 2.67 to 2.78 with *T. suis* intestine, and from 2.24 to 2.36 with *T. suis* esophagus. The LSVs observed for each sample appeared higher when they were compared with their corresponding body part (esophagus or intestine), but with both could be correctly identified.

#### Analysis of Samples for External Database Validation

During the study, a total of 20 *Trichuris* worms were utilized for blind testing 20 esophagus and 20 intestines for MALDI-TOF analysis. Of the 40 samples, two esophagus samples were not successfully extracted.

By using the newly developed internal database and considering a threshold of 1.70, MALDI-TOF MS correctly identified 38/38 (100%) of the samples at the probable genus level. When an LSV threshold of ≥2.0 was obtained, the identification rate was 9/18 (50%) for esophagus *T. suis* samples while for the intestine samples it was 16/20 (80%) for a probable species identification. Lastly, LSV ≥ 2.3 was 1/18 (5.55%) for esophagus samples and 1/20 (5%) for intestine samples with a highly probable species identification (see Table 1).

**Table 1:** Identification of 38 *T. suis* samples by MALDI-TOF MS, using a preliminary developed internal database.

**Species/body part (sample type)**	**Number of samples correctly extracted**	**Identification**			**LSV range**
		**LSV ≥1.70**	**LSV ≥2.00**	**LSV ≥2.30**	
*Trichuris suis*/Esophagus	18	18 (100%)	9 (50%)	1 (5.55%)	1.84–2.36
*Trichuris suis*/Intestine	20	20 (100%)	16 (80 %)	1 (5%)	1.90–2.34

*LSV, log-score value*.

### Validation of MALDI-TOF Analysis

To provide a molecular identification, 19 *cyt*b mtDNA partial sequences were assessed, of which the intestines of the whipworms were used, and were tested against previously deposited sequences by BLASTn, demonstrating high values of identity (98.81–100%) (see Supplementary Table S1).

**Figure 3:**
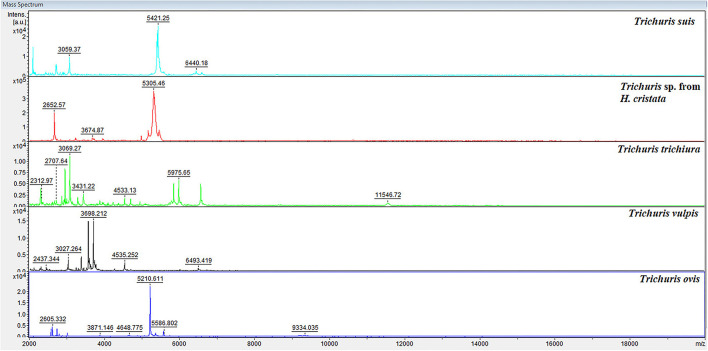
Representative spectral profiles of the esophagus of *Trichuris* species acquired for the creation of the internal database. a.u., arbitrary units; *m*/*z*, mass-to-charge ratio.

The preliminary protocol used for *Trichuris* samples preparation provided high-quality spectra with elevated reproducibility and intensity of MS spectra. To observe the characteristic high-intensity peaks, a representative protein spectral profile for each *Trichuris* species (*T. suis, Trichuris* sp. from *H. cristata, T. trichiura, T. vulpis*, and *T. ovis*) is shown in Figure 3. Hence, for each *Trichuris* species, specific and reproducible MALDI-TOF MS spectra profiles were obtained. Moreover, the internal database was updated with four new *Trichuris* species, and 16 new reference spectra: *Trichuris* sp. from *H. cristata* (*n* = 5), *T. vulpis* (*n* = 2), *T. ovis* (*n* = 4), and *T. trichiura* (*n* = 5).

When submitting the new MSPs of *Trichuris* species to the in-house database (combined with those data commercially available), all the 79 specimens used in the blind test were successfully queried in the extended database. The blind test results yielded 100% correct identification for the specimens evaluated since all samples were LSVs greater than 1.70 (Table 2). For the blind test, the percentage of samples with LSVs ≥ 2.00 was for *T. trichiura* from *M. sylvanus*: 88.89%; for *Trichuris* sp. from *H. cristata*: 72.22%; for *T. ovis*: 100%; and for *T. vulpis*: 87.5% (Table 2). Finally, the blind test confirmed a complete agreement with the molecular and MALDI-TOF MS identification methods.

**Table 2:** Identification of *Trichuris* species esophagus samples by MALDI-TOF MS, using an internal database.

**Species**	**Number of samples correctly extracted**	**Identification**							**LSV range**
		**LSV <1.7**	**LSV 1.7–1.799**	**LSV 1.8–1.999**	**LSV 2–2.299**	**LSV ≥2.3**	**LSV ≥1.70**	**LSV ≥2.00**	
*Trichuris trichiura* from *M. sylvanus*	18	0	0	2 (11.11%)	13 (72.22%)	3 (16.67%)	18 (100%)	16 (88.89%)	1.89–2.38
*Trichuris* sp. from *H. cristata*	18	0	1 (5.55%)	4 (22.22%)	12 (66.67%)	1 (5.55%)	18 (100%)	13 (72.22%)	1.72–2.42
*Trichuris ovis*	19	0	0	0	1 (5.26%)	18 (94.74%)	19 (100%)	19 (100%)	2.185–2.577
*Trichuris vulpis*	8	0	0	1 (12.5%)	5 (62.5%)	2 (25%)	8 (100%)	7 (87.5%)	1.95–2.52

*LSV, log-score value*.

**Figure 4:**
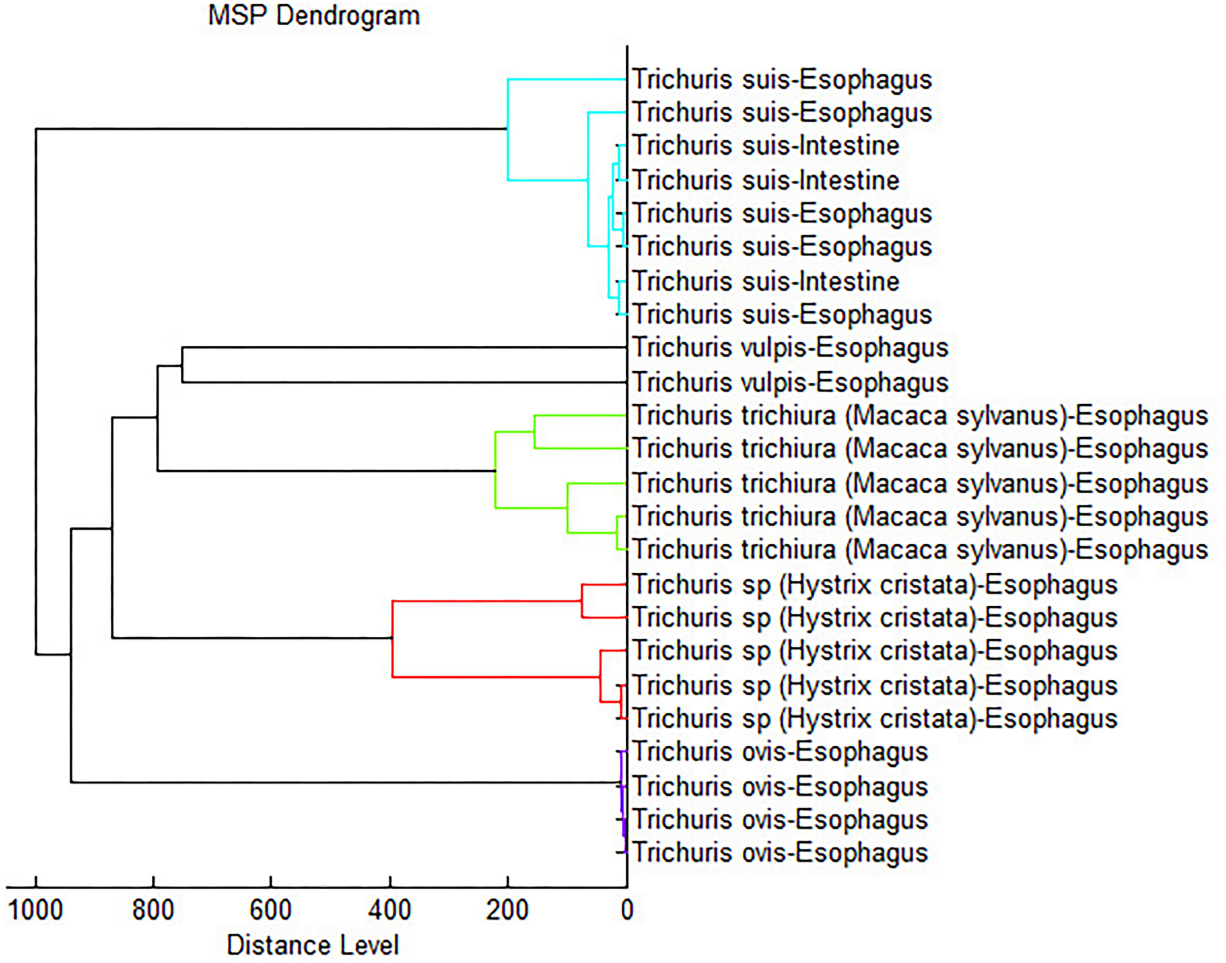
Dendrogram analysis constructed using representative spectra of each *Trichuris* species used to develop the internal database.

Furthermore, to confirm the accurate analysis, a dendrogram based on MSPs was added to the internal database with the five *Trichuris* species (including *T. suis*), and all species provided highly specific spectra with separated clades for each species (Figure 4).

### Molecular Study

The accession number of all nucleotide sequence data obtained in this work were deposited at the GenBank^TM^, EMBL, and DDBJ databases, and are available in Table 3.

**Table 3:** Summary of the *Trichuris* sequences obtained in this work and available in GenBank database.

**Species**	**Host**	**Sample ID**	**Accession number**	**Sequence length**	**Content of G + C%**
*Trichuris* sp.	*Hystrix cristata*	THCM1	OM457227	519	27.2
*Trichuris* sp.	*Hystrix cristata*	THCM2	OM457228	519	27.2
*Trichuris* sp.	*Hystrix cristata*	THCF1	OM457229	519	25.1
*Trichuris* sp.	*Hystrix cristata*	THCF2	OM457230	519	24.9
*Trichuris* sp.	*Hystrix cristata*	THCF3	OM457231	519	27.2
*Trichuris vulpis*	*Canis lupus familiaris*	TVM1	OM457232	519	28.5
*Trichuris vulpis*	*Canis lupus familiaris*	TVF1	OM457233	519	28.3
*Trichuris ovis*	*Capra hircus*	TOF1	OM457234	519	31.4
*Trichuris ovis*	*Capra hircus*	TOF2	OM457235	519	31.6
*Trichuris ovis*	*Capra hircus*	TOM1	OM457236	519	31.4
*Trichuris ovis*	*Capra hircus*	TOM2	OM457237	519	31.4
*Trichuris trichiura*	*Macaca sylvanus*	TMSF1	OM457238	519	30.7
*Trichuris trichiura*	*Macaca sylvanus*	TMSF2	OM457239	519	30.7
*Trichuris trichiura*	*Macaca sylvanus*	TMSF3	OM457240	519	31.1
*Trichuris trichiura*	*Macaca sylvanus*	TMSM1	OM457241	519	31.1
*Trichuris trichiura*	*Macaca sylvanus*	TMSM2	OM457242	519	30.7
*Trichuris suis*	*Sus scrofa domestica*	TSM1	OM457243	519	28.7
*Trichuris suis*	*Sus scrofa domestica*	TSF1	OM457244	519	28.9
*Trichuris suis*	*Sus scrofa domestica*	TSF2	OM457245	519	28.9

To compare the intraspecific and interspecific similarities obtained among *Trichuris* species, pairwise nucleotide and amino acid distances for the *cyt*b partial gene sequences were performed (Table 4). The intraspecific similarity between all the samples of the same species studied was similar with values close to 100% (Table 4), except for the intraspecific similarity among *Trichuris* sp. from *H. cristata*, which showed two different groups. The first group consisted of THCM1, THCM2, and THCF3 samples, and the second group of THCF1 and THCF2 samples, with intraspecific nucleotide similarity values of 100 and 99.8%, respectively, and 100% for amino acid distances. The interspecific nucleotide similarity between both groups was 88.2–88.4%, and amino acid similarity was 90.8%. The minimum interspecific similarity among all *Trichuris* species obtained was 68% (between *T. trichiura* from *M. sylvanus* and *Trichuris* sp. from *H. cristata*) in nucleotide distances, and the maximum was 73.6% (between *T. ovis* and *Trichuris* sp. from *H. cristata*) and the minimum in amino acid distances was 65.3% (between *T. trichiura* from *M. sylvanus* and *Trichuris* sp. from *H. cristata*) and the maximum was 76.3% (between *T. trichiura* from *M. sylvanus* and *T. suis*) (Table 4).

**Table 4:** Pairwise nucleotide and amino acid distances for the *cytochrome* b partial gene sequences for *Trichuris* species studied in this work.

	**THCM1**	**THCM2**	**THCF1**	**THCF2**	**THCF3**	**TVM1**	**TVF1**	**TOF1**	**TOF2**	**TOM1**	**TOM2**	**TMSF1**	**TMSF2**	**TMSF3**	**TMSM1**	**TMSM2**	**TSM1**	**TSF1**	**TSF2**
THCM1		100	90.8	90.8	100.0	68.2	68.2	68.8	68.8	68.8	68.8	65.3	65.3	65.9	65.3	65.3	69.4	69.4	69.4
THCM2	100.0		90.8	90.8	100.0	68.2	68.2	68.8	68.8	68.8	68.8	65.3	65.3	65.9	65.3	65.3	69.4	69.4	69.4
THCF1	88.4	88.4		100.0	90.8	68.8	68.8	69.4	69.4	69.4	68.8	65.9	65.9	66.5	65.9	65.9	69.9	69.9	69.9
THCF2	88.2	88.2	99.8		90.8	68.8	68.8	69.4	69.4	69.4	68.8	65.9	65.9	66.5	65.9	65.9	69.9	69.9	69.9
THCF3	100.0	100.0	88.4	88.2		68.2	68.2	68.8	68.8	68.8	68.8	65.3	65.3	65.9	65.3	65.3	69.4	69.4	69.4
TVM1	70.7	70.7	73.2	73.2	70.7		99.4	71.7	71.7	71.7	71.7	72.8	72.8	71.7	72.8	72.8	69.9	69.9	69.9
TVF1	70.7	70.7	73.2	73.2	70.7	99.8		71.7	71.7	71.7	71.7	72.8	72.8	71.7	72.8	72.8	69.9	69.9	69.9
TOF1	71.7	71.7	73.6	73.6	71.7	69.9	69.9		100.0	100.0	99.4	72.3	72.3	71.1	72.3	72.3	73.4	73.4	73.4
TOF2	71.5	71.5	73.4	73.4	71.5	69.7	69.7	99.8		100.0	99.4	72.3	72.3	71.1	72.3	72.3	73.4	73.4	73.4
TOM1	71.5	71.5	73.4	73.4	71.5	69.7	69.7	99.2	99.4		99.4	72.3	72.3	71.1	72.3	72.3	73.4	73.4	73.4
TOM2	71.5	71.5	73.2	73.2	71.5	69.7	69.7	99.2	99.4	98.8		72.8	72.8	71.7	72.8	72.8	73.4	73.4	73.4
TMSF1	68.2	68.2	69.4	69.6	68.2	70.3	70.5	70.1	69.9	69.7	70.1		100.0	98.8	100.0	100.0	76.3	76.3	76.3
TMSF2	68.2	68.2	69.4	69.6	68.2	70.3	70.5	70.1	69.9	69.7	70.1	100.0		98.8	100.0	100.0	76.3	76.3	76.3
TMSF3	68.2	68.2	69.4	69.6	68.2	69.9	70.1	69.7	69.6	69.4	69.7	99.6	99.6		98.8	98.8	76.3	76.3	76.3
TMSM1	68.0	68.0	69.2	69.4	68.0	70.1	70.3	70.1	69.9	69.7	70.1	99.6	99.6	99.2		100.0	76.3	76.3	76.3
TMSM2	68.2	68.2	69.4	69.6	68.2	70.3	70.5	70.1	69.9	69.7	70.1	100.0	100.0	99.6	99.6		76.3	76.3	76.3
TSM1	71.1	71.1	72.1	72.1	71.1	72.1	72.1	72.1	71.9	72.1	71.9	71.1	71.1	71.1	70.9	71.1		100.0	100.0
TSF1	70.9	70.9	71.9	71.9	70.9	71.9	71.9	72.1	71.9	72.1	71.9	70.9	70.9	70.9	70.7	70.9	99.8		100.0
TSF2	70.9	70.9	71.9	71.9	70.9	71.9	71.9	72.1	71.9	72.1	71.9	70.9	70.9	70.9	70.7	70.9	99.8	100.0	

*Nucleotide genetics distances are given below the diagonal and amino acid genetic distance above the diagonal*.

**Figure 5:**
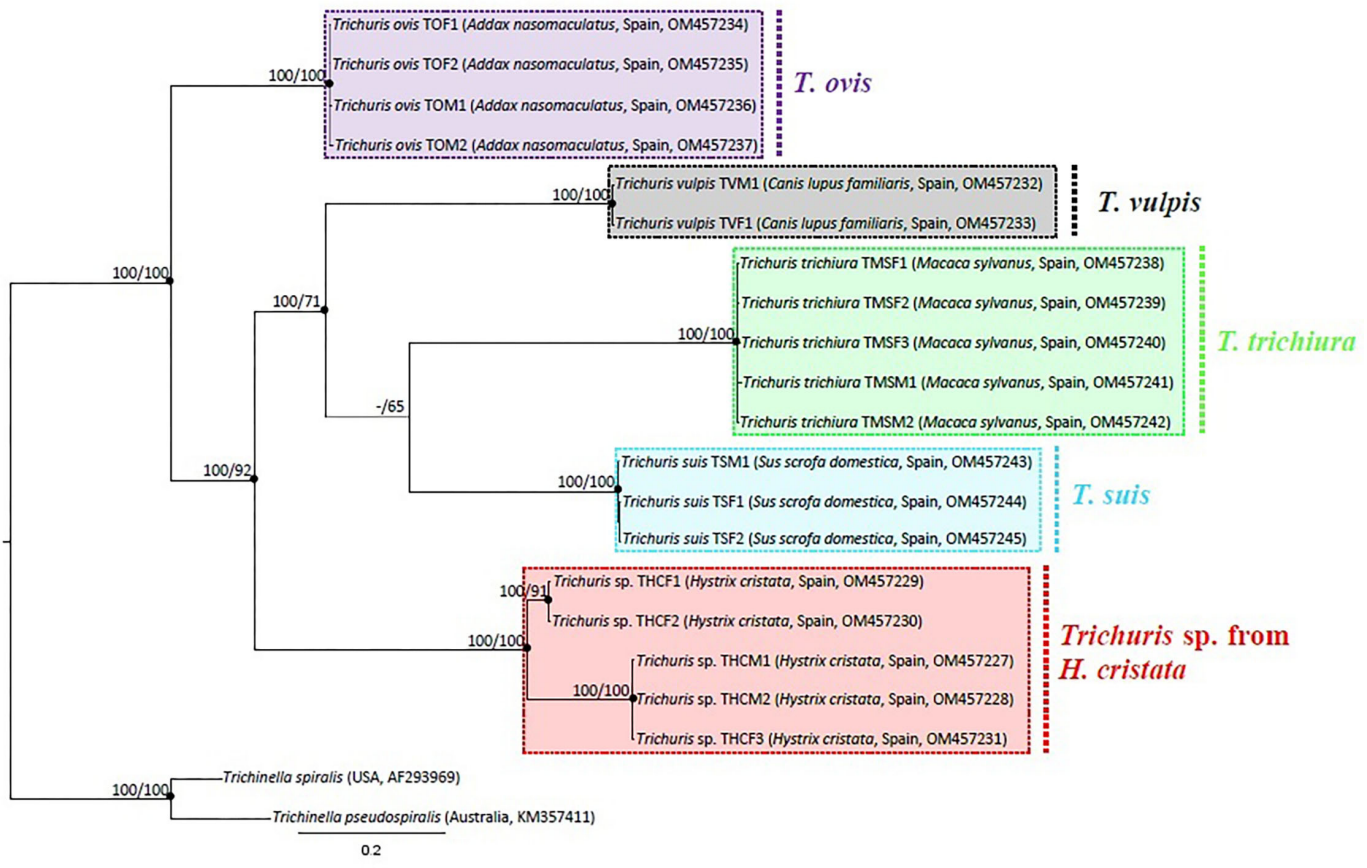
Phylogenetic tree based on analysis of mtDNA *cyt*b partial gene using Bayesian Inference. Maximum likelihood bootstrap values are listed first, followed by Bayesian Posterior Probabilities of clades, for clade frequencies exceeding 60%.

Phylogenetic tree based on *cyt*b sequences, was rooted including outgroup *Trichinella spiralis* and *Trichinella pseudospiralis*. The alignment of 20 sequences of *Trichuris* species (including outgroups), yielded a dataset of 505 characters. The phylogenetic tree revealed five main clades, corresponding each clade with one different species of *Trichuris*. All clades separately were highly supported (100% ML and 100% BPP). In addition, the clades *T. suis* and *T. trichiura* are more related to each other, but this relation is only supported by BPP, since there is a bootstrap value of <60% between the clades in ML. Both clades are, at the same time, more related to *T. vulpis*. And all the above is more related to *Trichuris* sp. from *H. cristata*, with the clade of *T. ovis* separated from all of them, although within the group of *Trichuris*. In addition, two different lineages, highly supported, among *Trichuris* sp. from *H. cristata* were revealed (Figure 5).

## Discussion

The genus *Trichuris* has been the subject of a wide-ranging controversy due to its difficult specific differentiation. Many authors have evidenced synonymies (62), cryptic species (18, 63), and new species (6, 7, 10, 64, 65). Since Dujardin (66) reviewed the genus of *Trichuris* for the first time, many studies have been based on morphometric and molecular analyses. The difficulty of relating the species described with exclusively morphological studies and with species described with only molecular studies makes the study of taxonomy even more difficult to solve. In support of this, additional complementary methods might be necessary to elucidate the different species.

The aim of this study was to confirm whether MALDI-TOF MS analysis could be used as a diagnostic tool for the identification of adult *Trichuris* species. Our results showed high-quality spectra and a high similarity among the samples, regardless of the type of sample analyzed, so we confirm the use of both parts of the body of the nematode for its diagnosis using this technique. To achieve this goal, an internal database has been developed with samples of both, the esophagus and intestine of *T. suis*, providing similar spectra between them, and, therefore, being adequate to identify this species. All specimens (38/38) were accurately identified. Moreover, the preliminary protocol was validated for more species of *Trichuris*, including four more species of *Trichuris* in the internal database. These *Trichuris* species were also 100% correctly identified. As the values revealed in the preliminary study of samples, both esophagus and intestines, showed similar results, we suggest the preference of using the worm's esophagus through MALDI-TOF MS technique since it is easier to manage the esophagus and saving the part of the worm's intestine for molecular studies.

MALDI-TOF MS has been used previously as an effective diagnostically tool in microbiology clinical to identify pathogenic microorganisms. Even now, a systematic review of about MALDI-TOF MS in human and veterinary helminthology was carried out, concluding that more studies are needed since there is evidence for the reliable and rapid identification of nematodes using MALDI-TOF MS, and the identification of these nematodes, whether larvae, adults, or eggs in fecal samples (49). Hence, the internal database for helminth identification is being generated with species-specific MSPs. It is necessary the advancement of one standardized approach for protein extraction and MALDI-TOF MS spectra to create accurate databases and specific and reproducible results in different laboratories for further research (25). Nonetheless, these internal databases are created in each reference laboratory, and for that reason, we find ourselves with the added difficulty of not being able to compare the different species recorded in the different databases created, being of huge importance to the creation of a single common database or being able to share the spectra obtained from the analyzes of each research laboratory.

Nagorny et al. (67), in their research for the identification of different nematodes (*Dirofilaria* and *Ascaris*) by MALDI-TOF MS using tissues from adult worms, suggested that in the range from 8 to 20 kDa, the spectra allowed differentiating between different species of nematodes, and in the range of 2 to 6 kDa, the entire genus of nematodes could probably be characterized. In our spectra obtained for the MSP, the most frequent peaks were observed in the range from 2 to 7 kDa but extended up to 10 kDa. Consequently, the necessity for the identification of more species of *Trichuris* to know whether really exist a differential range for genus and species of nematodes in the spectra obtained by MALDI-TOF MS in further research.

Furthermore, MALDI-TOF MS technique has been advanced in the last few years as a significant tool for taxonomic identification and for the phylogenetic classification of microorganisms (68). Subsequently, Zurita et al. (22) supported the evidence with flea vector species, obtaining agreement with the data obtained in the dendrogram and the phylogenetic studies carried out. Nevertheless, those authors in agree with Yssouf et al. (69), argued that due to the lack of specimens, MALDI Biotyper software cannot yet determine reliablility for the phylogenetic study of arthropods. Likewise, studies of parasitic nematodes suggest the usefulness of MALDI-TOF as an efficient taxonomic tool in parasitological studies (67). In this study, the obtained dendrogram separated each *Trichuris* species into a different clade. Moreover, it related more to the clades of *T. vulpis* and *T. trichiura*, and on the other hand, the clades of *T. suis* and *Trichuris* sp. from *H. cristata*, resulting in the clade of *T. ovis* more separated from all other clades. This last argument is the same as the one found in the phylogenetic tree based on *cyt*b mtDNA partial gene, where *T. ovis* clade is always separated from the other clades; however, the phylogenetic relationships among the other clades are different and were highly supported. Thus, according to previously described authors, we suggest that MALDI-TOF MS should not be used to establish phylogenetic relationships in nematode species, but further studies are needed with more different species and genera of nematode parasites.

Soil-transmitted helminth (STH) infections are among the most common infections worldwide, affecting the poorest and most disadvantaged communities. The global strategy for controlling the morbidity of STH infections is preventive chemotherapy with periodic medicinal treatment (deworming) without a previous individual diagnosis for all at-risk people living in endemic areas (preschool and school-age children and women of reproductive age and adults in certain high-risk occupations). The medicines recommended by WHO are albendazole or mebendazole, which are effective, inexpensive, and easy to administer by non-medical personnel (1). Both drugs show low efficacy against *T. trichiura* using single, oral doses. Frightening, according to a recent network meta-analysis looking at interactions over time, the efficacy of both drugs is declining over time, which could be associated with resistance to anthelmintic drugs (70). Moreover, MALDI-TOF MS analysis is used as a tool to discover the antibiotic resistance in microorganisms by detection of precise biomarkers within the protein spectra produced (46, 47, 71), and could be used to find different targets and to develop treatments to combat the resistance. A preliminary study about a new diagnostic tool for *Anisakis* spp. by MALDI-TOF MS, has managed to identify a set of signs as potential consensus “biomarkers” peak list (24). In this study, we started by characterizing a genus and a species that has never been determined by MALDI-TOF analysis and developing the first internal database with *Trichuris* nematode parasites. But more *Trichuris* species would be needed to differentiate between their protein spectra profile and draw conclusions.

## Conclusion

This study validates the usefulness of MALDI-TOF MS technique as a reliable, fast, and economical identification tool for the diagnosis of *Trichuris* species. The creation of the internal database should be expanded with more samples of different species of the genus *Trichuris* and other nematodes species. The results obtained by MALDI-TOF MS showed a dendrogram that is not reliable to phylogenetic studies in *Trichuris* species. In addition, the necessity to discover and analyze potential biomarkers and targets to focus future studies on developing new anthelmintic drugs.

## Data Availability Statement

The raw data supporting the conclusions of this article will be made available by the authors, without undue reservation.

## Author Contributions

JR, AZ, and RC: conceptualization and design of the study, investigation, validation, and methodology. JR: data curation and writing–original draft. JR and AZ: formal analysis. CC: funding acquisition. RC and CC: project administration and supervision. All authors contributed to the manuscript revision, read, and approved the submitted version.

## Funding

This work was financially supported by a Grant (CGL2017-83057) funded by MCIN/AEI/10.13039/501100011033 and by ERDF A way of making Europe.

## Conflict of Interest

The authors declare that the research was conducted in the absence of any commercial or financial relationships that could be construed as a potential conflict of interest.

## Publisher's Note

All claims expressed in this article are solely those of the authors and do not necessarily represent those of their affiliated organizations, or those of the publisher, the editors and the reviewers. Any product that may be evaluated in this article, or claim that may be made by its manufacturer, is not guaranteed or endorsed by the publisher.
