# Supplementary material for: The Use of MALDI-TOF MS as a Diagnostic Tool for Adult Trichuris Species
Source: Front Vet Sci. 2022 May 11;9:867919. doi: 10.3389/fvets.2022.867919 (PMC9132177; doi:10.3389/fvets.2022.867919)
Supplement: Supplementary file 1 [file Table_1.DOCX]

**Table S1.** Homology values ​​obtained from comparing the *cyt*b sequences of the different species of *Trichuris* obtained in the present work, with previously described sequences deposited in NCBI GenBank.

| **Sample ID** | **Species** | **Host** | **Reference accession number** | **Percent Identity (%)** |
| --- | --- | --- | --- | --- |
| TSM1 | *Trichuris suis* | *Sus scrofa domestica* | LM994696, KT449822 | 98.81 |
| TSF1 | *Trichuris suis* | *Sus scrofa domestica* | LM994696, KT449822 | 100 |
| TSF2 | *Trichuris suis* | *Sus scrofa domestica* | LM994696, KT449822 | 100 |
| THCM1 | *Trichuris* sp. | *Hystrix cristata* | OU596148 | 100 |
| THCM2 | *Trichuris* sp. | *Hystrix cristata* | OU596148 | 100 |
| THCF1 | *Trichuris* sp. | *Hystrix cristata* | OU596147 | 99.81 |
| THCF2 | *Trichuris* sp. | *Hystrix cristata* | OU596147 | 100 |
| THCF3 | *Trichuris* sp. | *Hystrix cristata* | OU596148 | 100 |
| TVM1 | *Trichuris vulpis* | *Canis lupus familiaris* | LM994699 | 99.81 |
| TVF1 | *Trichuris vulpis* | *Canis lupus familiaris* | LM994699 | 100 |
| TOF1 | *Trichuris ovis* | *Capra hircus* | LM994697 | 99.61 |
| TOF2 | *Trichuris ovis* | *Capra hircus* | LM994697 | 99.81 |
| TOM1 | *Trichuris ovis* | *Capra hircus* | LM994697 | 99.23 |
| TOM2 | *Trichuris ovis* | *Capra hircus* | LM994697 | 99.23 |
| TMSF1 | *Trichuris trichiura* | *Macaca sylvanus* | MW448472, MW448470, KT449826 | 100 |
| TMSF2 | *Trichuris trichiura* | *Macaca sylvanus* | MW448472, MW448470, KT449826 | 100 |
| TMSF3 | *Trichuris trichiura* | *Macaca sylvanus* | MW448472, MW448470, KT449826 | 99.61 |
| TMSM1 | *Trichuris trichiura* | *Macaca sylvanus* | MW448472, MW448470, KT449826 | 99.61 |
| TMSM2 | *Trichuris trichiura* | *Macaca sylvanus* | MW448472, MW448470, KT449826 | 100 |
